# Localized genital blisters in an elderly male

**DOI:** 10.1016/j.jdcr.2025.01.032

**Published:** 2025-03-03

**Authors:** An Jian Leung, Dingyuan Wang

**Affiliations:** a Department of Medicine, National University Hospital, Singapore; b National Skin Centre, Singapore

**Keywords:** autoimmune, blisters, bullous pemphigoid, genital, vesicle

A 91-year-old Chinese male presented with a 1-week history of genital blisters noted by his caregiver. He had Parkinsonism, diabetes mellitus, hypertension, chronic kidney disease, gout, and a previous aortic valve repair. Physical examination revealed scrotal and perineal vesiculobullae with scattered superficial erosions (Fig 1). He was otherwise well and the rest of his body, including mucous membranes, were unaffected. He received oral amoxicillin and topical steroids with little relief prior to specialist review. There were no new contactants. He was dually continent and ambulatory with a quad-stick. Full blood count, inflammatory markers, liver, and renal function tests were at baseline.

**Fig 1:**
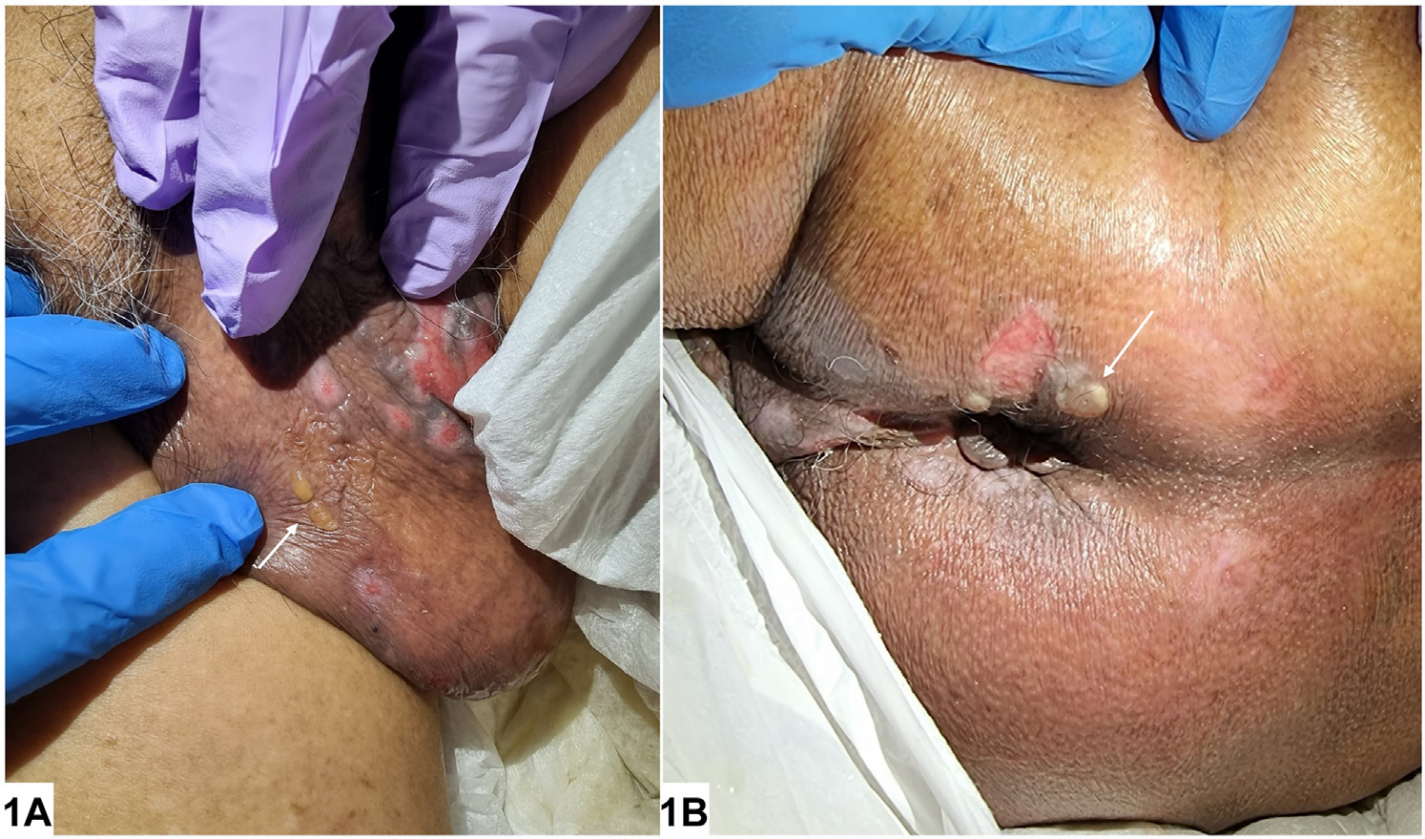

**Question 1: What is the most likely diagnosis?**

A. Bullous impetigo
B. Bullous pemphigoid (BP)
C. Bullous tinea cruris
D. Dermatitis herpetiformis
E. Varicella zoster infection

**Answers:**

A. Bullous impetigo – Incorrect. Classic honey-golden crusted lesions with systemic symptoms like fever and lethargy expected in bullous impetigo were not observed here, making this less likely. Furthermore, little relief with amoxicillin was noted.
B. Bullous pemphigoid (BP) – Correct. Localized genital bullous pemphigoid is a rare clinical variant of BP. It tends to affect children or females with only a handful of reports in elderly males.^1^ Clinically, vesicles and bulla with superficial erosions are such characteristics. Typical urticated plaques and erythema were less evident in this case since they appeared on thickened and hyperpigmented scrotal skin.
C. Bullous tinea cruris – Incorrect. The lack of an annular erythematous plaque with an inflammatory border and the sparing of skin folds makes fungal infection less likely. Fungal smears returned negative in this case.
D. Dermatitis herpetiformis – Incorrect. Typically appearing as symmetrical vesicles and blisters on the elbows, sacrum and buttocks, dermatitis herpetiformis is a subepidermal immunobullous disorder that can be associated with underlying celiac disease. While sacral lesions are typical, the presence of scrotal blisters, advanced age at diagnosis, male gender and the lack of reported gastrointestinal sensitivity to gluten makes this diagnosis less likely.
E. Varicella zoster infection – Incorrect. While crops of vesicles may mimic localized genital bullous pemphigoid, multidermatomal varicella zoster virus crossing the midline and localized to the groin in this pattern is highly unusual. Neuropathic symptoms like tingling or pain (not observed in our patient) would also feature prominently. Varicella zoster virus and herpes simplex DNA polymerase chain reaction returned negative in this case.

**Question 2: Which of the following are NOT associated with the diagnosis?**

A. Bedridden condition
B. Cerebrovascular disease
C. Chronic kidney disease
D. Male sex
E. Parkinson’s disease

**Answers:**

A. Bedridden condition – Incorrect. Bedridden condition and advanced age are associated with BP in prospective case-control studies.^2^^,^^3^
B. Cerebrovascular disease – Incorrect. Neurological disorders like stroke, multiple sclerosis, Shy-Drager syndrome, epilepsy and amyotrophic lateral sclerosis are associated with BP.^2^
C. Chronic kidney disease – Incorrect. Chronic kidney disease is associated with increased incidence and mortality in BP.^2^^,^^3^
D. Male sex – Correct. A female preponderance in observed in genital BP. It should, however, be noted that a male preference is seen in ordinary BP.^2^^,^^3^
E. Parkinson’s disease – Incorrect. Parkinson’s disease is associated with BP. Psychiatric disorders associated with BP include schizophrenia and delusional disorders. While no pathogenic basis has been credibly established, one mechanism postulated is the production of anti-BP180 and anti-BP230 in response to damaged brain tissue resulting in basement membrane antigen cross reactivity and blister formation.^2^^,^^3^ Neoplastic evaluation should also be undertaken in elderly-onset autoimmune blistering diseases to exclude an underlying malignancy.

**Question 3: What serological markers can confirm the diagnosis?**

A. Anti-BP 180 and 230
B. Anticollagen type VII
C. Antidesmoglein 1 and 3
D. Antienvoplakin
E. Anti-p200

**Answers:**

A. Anti-BP 180 and 230 – Correct. Anti-BP 180 and BP 230 are diagnostic serological markers in BP.^4^ Both were positive in our case with positive serum indirect immunofluorescence on salt-split skin (titres 1:160) in a roof pattern. He received lesional mometasone ointment 0.1% with modest effect. Flaccid blisters subsequently erupted over the arms, palms and feet a month later. A skin biopsy over the hand showed eosinophilic spongiosis with dermal eosinophilia and linear C3 deposits along the basement zone, consistent with BP. Localized genital bullous pemphigoid with secondary generalization was hence diagnosed. Treatment was escalated to whole body ultrapotent topical steroids, oral prednisolone, doxycycline, and nicotinamide with good effect.
B. Anticollagen type VII – Incorrect. This antibody is present in epidermolysis bullosa acquisita and bullous systemic lupus erythematosus.^4^
C. Antidesmoglein 1 and 3 – Incorrect. This antibody is present in intraepidermal blistering diseases like pemphigus vulgaris, pemphigus foliaceus, immunoglobulin A pemphigus, drug-induced pemphigus, and pemphigus herpetiformis.^4^
D. Antienvoplakin – Incorrect. This antibody is present in paraneoplastic pemphigus.^4^
E. Anti-p200 – Incorrect. This antibody is present in antilaminin γ1/p200 pemphigoid.^4^

## Conflicts of interest

None disclosed.
